# Notice of Stockton's Establishment

**Published:** 1841

**Authors:** 


*Stockton's establishment for Teeth, Dental Instruments, &c. 243 Broadway, New-York.*

We would invite the attention of the profession to the above establishment, where, we understand, is constantly kept every article used by the dentist in his practice—such as Porcelain Teeth, Dental Instruments of every description, including every variety of files—an article, by the by, not always easy to procure—Gold Foil, Plate, Springs, &c., Operating Chairs, Desks, and every other fixture needed by the practitioner in this department of Surgery.

The difficulty of obtaining many of the above articles, has been experienced by almost every member of the profession ; we are therefore gratified that so indefatigable a man as Mr. Stockton, in whatever he undertakes, has opened an establishment of this kind. It will be a great accommodation to the profession, and we doubt not, will be profitable to the proprietor. To the list of articles which Mr. S. proposes to keep, we would suggest the propriety of adding all the standard works on dental surgery.

